# Selective Inhibitor of Nuclear Export (SINE) Compounds Alter New World Alphavirus Capsid Localization and Reduce Viral Replication in Mammalian Cells

**DOI:** 10.1371/journal.pntd.0005122

**Published:** 2016-11-30

**Authors:** Lindsay Lundberg, Chelsea Pinkham, Cynthia de la Fuente, Ashwini Brahms, Nazly Shafagati, Kylie M. Wagstaff, David A. Jans, Sharon Tamir, Kylene Kehn-Hall

**Affiliations:** 1 National Center for Biodefense and Infectious Diseases, School of Systems Biology, George Mason University, Manassas, Virginia, United States of America; 2 Nuclear Signaling Laboratory, Department of Biochemistry and Molecular Biology, Monash University, Clayton, Victoria, Australia; 3 Karyopharm Therapeutics Inc., Massachusetts, United States of America; Centers for Disease Control and Prevention, UNITED STATES

## Abstract

The capsid structural protein of the New World alphavirus, Venezuelan equine encephalitis virus (VEEV), interacts with the host nuclear transport proteins importin α/β1 and CRM1. Novel selective inhibitor of nuclear export (SINE) compounds, KPT-185, KPT-335 (verdinexor), and KPT-350, target the host’s primary nuclear export protein, CRM1, in a manner similar to the archetypical inhibitor Leptomycin B. One major limitation of Leptomycin B is its irreversible binding to CRM1; which SINE compounds alleviate because they are slowly reversible. Chemically inhibiting CRM1 with these compounds enhanced capsid localization to the nucleus compared to the inactive compound KPT-301, as indicated by immunofluorescent confocal microscopy. Differences in extracellular versus intracellular viral RNA, as well as decreased capsid in cell free supernatants, indicated the inhibitors affected viral assembly, which led to a decrease in viral titers. The decrease in viral replication was confirmed using a luciferase-tagged virus and through plaque assays. SINE compounds had no effect on VEEV TC83_Cm, which encodes a mutated form of capsid that is unable to enter the nucleus. Serially passaging VEEV in the presence of KPT-185 resulted in mutations within the nuclear localization and nuclear export signals of capsid. Finally, SINE compound treatment also reduced the viral titers of the related eastern and western equine encephalitis viruses, suggesting that CRM1 maintains a common interaction with capsid proteins across the New World alphavirus genus.

## Author Summary

Our data demonstrate that novel selective inhibitor of nuclear export (SINE) compounds reduced viral replication of three related New World alphaviruses, VEEV, EEEV, and WEEV, indicating that CRM1 is instrumental to their life cycle. The novel CRM1 inhibitors have a large selective index and represent a potential pan-antiviral therapeutic that targets the host’s transport proteins, which are hijacked by the New World alphaviruses.

## Introduction

Endemic to North, Central, and South America, the New World alphaviruses cause a febrile illness that can progress to encephalitis with accompanying high morbidity and mortality rates in humans and equines [1]. Three viruses in particular, Venezuelan, western, and eastern equine encephalitis viruses (VEEV, WEEV, and EEEV), are of concern both as naturally emerging infectious diseases and potential bioweapons [2]. There are currently no FDA-approved antivirals or vaccines for use in humans. Considerable research effort has been aimed at studying the less pathogenic Old World alphaviruses like Sindbis Virus (SINV), creating a gap in knowledge of pathogenesis and therapeutic targets of New World alphaviruses [3].

Alphaviruses belong to the single-stranded, positive sense RNA family *Togaviridae*. Alphaviruses are divided into New World and Old World groups based on geography, disease progression, and protein function. Severe cases of Old World alphavirus infections present with arthritic symptoms, while New World infections often become encephalitic [4]. The alphavirus genome has two distinct regions. The nonstructural region encodes a polyprotein from the genomic RNA that is cleaved into the nonstructural proteins 1–4 (nsP1-4). The structural region codes a polyprotein from the subgenomic mRNA that is cleaved into the capsid protein, envelope glycoproteins, and several other small polypeptides [5]. The functional role of the viral proteins diverges between Old and New World alphaviruses. Type I interferons (IFN), IFN-α and IFN-β, attenuate alphaviruses, and Old and New World viruses have evolved to counter the host immune response. The VEEV nonstructural proteins inhibit STAT1 activation, preventing its nuclear localization and STAT1-dependent transcription [6]. The nsP2 protein of the Old World alphavirus Chikungunya (CHIKV) inhibits STAT1 activation by blocking its phosphorylation [7]. Binding to heparin sulfate alters the cellular tropism of EEEV, allowing it to evade the host immune responses [8]. Other mechanisms involve shutting down cellular transcription globally [2]. In SINV infection, host transcription downregulation is modulated by nsP2, a critical component of the virus’ replicative enzyme complex [9]. Conversely, VEEV, as a model for the New World viruses, uses its capsid protein to halt host transcription [10]. Capsid most likely achieves this by blocking nuclear import and export [11].

The nuclear pore is a complex macromolecular gate, regulating the nuclear entry and exit of most macromolecules including proteins >40 kDa. Transit through the pore requires carrier molecules called importins and exportins. Both nuclear transport processes are highly regulated and localization within the cell is an important mechanism for regulating macromolecular function. The major human nuclear export protein is exportin 1 (XPO1, also called chromosome region maintenance 1, CRM1). CRM1 mediates the nuclear export of over 200 proteins with canonical nuclear export sequences [12], along with some proteins that use adapter molecules and a small number of RNA transcripts. CRM1, plays a role in the life cycle of several viruses, such as HIV [13], dengue virus [14], and influenza virus [15, 16], and chemically targeting it has potential as an antiviral therapeutic. By forming a tetrameric complex with the host transport proteins CRM1 and importin α/β, VEEV capsid protein disrupts functioning of the nuclear pore complex [17]. Moreover, siRNA mediated knockdown of CRM1 resulted in confinement of VEEV capsid to the nucleus, whereas loss of importin α and/or importin β resulted in VEEV capsid being primarily cytoplasmic [18]. It is assumed that the capsid of EEEV, WEEV, and other New World alphaviruses behave similarly due to the phylogenic similarity of their capsid proteins [19]. Additionally, it has been demonstrated that the capsid protein of EEEV also inhibits host transcription [20] in mammalian, but not mosquito cells [21], similar to VEEV.

Our group previously investigated disruption of the VEEV capsid/host protein complex by chemically inhibiting host nuclear import and export proteins. Targeting the nuclear pore complex with the aim of inhibiting viral replication has been proposed by others [22, 23]. Inhibitors previously demonstrated to modulate viral protein binding to importin α and β1, mifepristone [24] and ivermectin [25], and the well-documented CRM1 inhibitor, Leptomycin B [26–28], altered the cellular distribution of VEEV capsid, reduced viral replication, and increased cell survival. As expected, mifepristone and ivermectin kept capsid primarily cytoplasmic, while Leptomycin B trapped capsid in the nucleus. Altering capsid localization reduced viral titers of both the VEEV vaccine strain, VEEV-TC83, and the wild-type strain, Trinidad Donkey (VEEV-TrD). Interestingly, inhibitor treatment also increased cellular viability in infected cells compared to infected cells treated with the vehicle only [18]. However, the traditional CRM1 inhibitor, Leptomycin B, is cytotoxic, creating the need for a new generation of CRM1 inhibitors.

Karyopharm Therapeutics has discovered potent, small molecule, orally available, slowly reversible inhibitors of CRM1, termed selective inhibitor of nuclear export (SINE) compounds. The most advanced drug in this class is selinexor, which is currently under clinical investigation for the treatment of patients with advanced hematologic and solid malignancies (ClinicalTrials.gov identifier: NCT01986348). After administration to over 1400 patients as of February 2016, selinexor (KPT-330) is showing preliminary evidence of durable anti-tumor activity in multiple cancer types. SINE compounds are well tolerated *in vitro* and *in vivo* [29, 30], and some of the compounds can cross the blood-brain barrier [12]. Due to previous successes in several viral models and their tolerability in human cancer clinical trials [31], three active compounds, referred to henceforth as KPT-185, KPT-335, and KPT-350, which are analogs of selinexor, and one inactive control compound, KPT-301, were tested for antiviral activity against three New World alphaviruses.

## Methods

### CRM1 inhibitors

The selective CRM1 inhibitors KPT-335, KPT-350, KPT-185, and the inactive trans-enantiomer KPT-301 were provided by Karyopharm Therapeutics (Newton, MA). KPT-185 was characterized most extensively in *in vitro* assays, but has poor PK properties unsuitable for use *in vivo*. KPT-335 (verdinexor) and -350 are less potent against CRM1 than KPT-185, but have similar specificity, good oral bioavailability and good tolerability, thereby are suitable for future *in vivo* studies. KPT-335 (verdinexor) has been tested in a Phase 1 healthy volunteer clinical trial and found to be safe and well-tolerated (ClinicalTrials.gov Identifier: NCT02431364). KPT-350 has a higher brain penetration ratio than KPT-335 (verdinexor).

### Cell culture

Vero cells and mouse embryonic fibroblasts (MEFs) were maintained as described previously [18]. BHK-J cells were maintained at 37°C, 5% CO_2_ in Eagle’s Minimum Essential Medium (EMEM) (ATCC, Manassas, VA, 30–2003) supplemented with 7.5% fetal bovine serum (FBS) and 1% penicillin/streptomycin.

### CRM1 inhibitor treatment

SINE compounds were dissolved in sterile DMSO, diluted in DMEM supplemented with 10% FBS, 1% penicillin/streptomycin and 1% L-glutamine, and incubated on cells for two hours prior to viral infection unless otherwise noted. Following infection, inhibitor-containing media was added back to the cells and remained for the duration of the experiment unless otherwise noted. Likewise, cells pre-treated with Leptomycin B at 45 nM prior to viral infection were also post-treated after infection, unless otherwise noted. Leptomycin B was purchased from Sigma Aldrich (L2913).

### Viruses and infections

VEEV-TC83 viral stocks were produced from electroporation of *in vitro* transcribed viral RNA generated from the pTC83 plasmid [a kind gift from Ilya Frolov, The University of Texas Medical Branch at Galveston [32, 33]]. In brief, the viral cDNA was linearized with *MluI* restriction enzyme (NEB) and purified using the Minelute PCR Purification kit (Qiagen) according to manufacturer’s directions. Capped RNAs were synthesized using the Sp6 MegaScript kit (Invitrogen) with a 2:1 ratio of cap analog (m7G(5')ppp(5')G [NEB]) to GTP and treated with DNase I supplied with the kit. RNA was then isolated with the RNeasy Mini kit with a second DNAse I on-column digestion (Qiagen). RNA integrity and concentration were determined by gel electrophoresis and absorbance at 260 nm, respectively.

*In vitro* transcribed viral RNA was electroporated into BHK-J cells (provided by Charles M. Rice) utilizing a 2 mm gap cuvette (BTX ECM 630 Exponential Decay Wave Electroporator; Harvard Apparatus, Holliston, MA). After trypsinization, cells were washed twice and resuspended in cold Dulbecco's phosphate-buffered saline without Ca^2+^/Mg^2+^ (RNase-free) at 1.25 × 10^7^ cells/ml. An aliquot of the cell suspension (400 μL) was mixed with 1 μg of RNA transcripts, placed into the cuvette, and pulsed once at 860 V, 25 μF capacitance, and 950 Ω resistance. Cells were then left to recover for 5 min at room temperature and resuspended in complete MEM media (Gibco-Invitrogen). Cells from three replicate electroporations were plated in three 75cm^2^ culture flasks for virus production. Next day [~12 hours post electroporation (hpe)], transfection media was replaced with fresh MEM media. At 18, 24 and 30 hpe, media supernatants were harvested, pooled and stored at 4° C. After the last collection, supernatants were then filtered (0.2 μM), aliquoted, and stored at -80°C. Viral titers were determined by plaque assay.

The VEEV-TC83luc virus was kindly provided by Dr. Slobodan Paessler of the University of Texas Medical Branch, Galveston [34]. VEEV-TC83 and SINV (EgAr 339), both obtained from BEI Resources, and VEEV-TC83luc, were utilized under BSL-2 conditions. VEEV-TrD, EEEV (strain GA97), WEEV (strain California 1930), and CHIKV (strain S27) were utilized under BSL-3 conditions. Working stocks were prepared by infecting Vero cells at an MOI 0.1 and collecting supernatants at 48 hours post-infection (hpi). Supernatants were clarified by centrifugation at 10,000xg for 10 minutes at 4°C to pellet cellular debris and then filtered through a 0.2 μM filter. Supernatants were aliquoted into 0.5 or 1 mL user stocks and titer determined by plaque assay. All work involving select agents is registered with the Centers for Disease Control and Prevention and conducted at George Mason University’s Biomedical Research Laboratory, which is registered in accordance with Federal select agent regulations.

When performing infections, virus was added to supplemented DMEM at a multiplicity of infection (MOI) of 1, unless otherwise stated. The viral media was added to cells and incubated at 37°C for one hour with rotation every fifteen minutes. After infection, cell cultures were washed once with sterile 1X PBS and media containing inhibitors was added back onto the cells unless noted otherwise.

Crystal violet plaque assays were used to determine viral titer as described previously [18].

### Cell viability assays

To determine inhibitor toxicity, Promega’s CellTiter Luminescent Cell Viability Assay (G7571) was used to measure cellular viability 24 hours after inhibitor treatment, as described previously [18].

### Immunofluorescence analysis

Vero cells were grown on cover-slips in a 6-well plate and processed for immunofluorescence analysis as described [18]. Antibodies used included anti-VEEV-capsid (BEI Resources, NR-9403) goat primary antibody (1:1000 dilution) and Alexa Fluor 568 donkey anti-goat secondary antibody (1:500 dilution). Slides were imaged using an oil-immersion 60X objective lens on a Nikon Eclipse TE 2000-U confocal microscope, with all samples subjected to four line averaging. At least three images were taken of each sample, with one representative image shown. The resulting images were processed through Nikon NIS-Elements AR Analysis 3.2 software. Digitized images were analyzed as previously described using the ImageJ version 1.47 public domain software (NIH) [18]. Briefly, Fn/c was calculated according to the formula Fn/c = (Fn-Fb)/(Fc-Fb), where Fn is the mean fluorescence determined from a typical region within the nuclei, Fc is the mean fluorescence of cytoplasm determined in the same cell from a region of cytoplasm close to the nucleus, and Fb is mean background autofluorescence determined from a mock infected sample, fixed and stained in the same fashion and imaged under the same conditions as the sample slide. See S1B Fig for an example.

### RNA extraction and quantitative RT-PCR

Supernatants were collected to analyze extracellular viral RNA and infected cells lysed and collected in Qiagen’s RLT buffer to analyze intracellular RNA. Extracellular viral RNA was extracted using Ambion’s MagMax Viral RNA Isolation Kit (AM1836); intracellular RNA was isolated using Qiagen’s RNeasy Mini Kit (74104). Q-RT-PCR was performed as described previously [35] for viral RNA using the Applied Biosystems StepOne Plus. Primer-pairs (forward TCTGACAAGACGTTCCCAATCA, reverse GAATAACTTCCCTCCGACCACA) and TaqMan probe (5′ 6-carboxyfluorescein-TGTTGGAAGGGAAGATAAACGGCTACGC-6-carboxy-*N*,*N*,*N*′,*N*′-tetramethylrhodamine-3′) against 7931–8005 of VEEV-TC83 were utilized [36]. The absolute quantification was calculated using StepOne Software v2.3 and based on the threshold cycle relative to the standard curve. The standard curve was established using serial dilutions of VEEV-TC83 RNA at known concentrations. To assay cellular genes, mRNA was converted to cDNA using the High Capacity RNA-to-cDNA kit (Life Technologies) according to the manufacturer’s protocol. Gene expression was assayed using the following TaqMan assays: IFIT1 (Mm00515153_m1), IFIT2 (Mm00492606_m1), IFNβ1 (Mm00439552_s1), and OASL1 (Mm00455081_m1). 18S ribosomal RNA (Mm04277571_s1) was used for normalization.

### Viral purification and protein quantification by western blot

Supernatants and whole cell lysates (WCL) were collected twenty-two hpi. The WCL were processed as described previously [18]. To purify viral proteins, the supernatants were subjected to sucrose density centrifugation. Briefly, supernatants were spun at 2,500 x g for 30 minutes to remove debris. 20%-60% continuous sucrose density gradients were prepared with supernatants layered on top. Samples were centrifuged at 36,000 RPM at 4°C for 4 hours. One milliliter fractions were collected and the third and fourth fractions were collected for analysis. WCL and purified supernatants in lysis buffer were separated on NuPAGE 4–12% Bis-Tris gels (Invitrogen) and transferred to PVDF as previously described [37]. The primary and secondary antibodies used were: anti-capsid (goat primary, BEI Resources, NR-9403), anti-actin (Abcam, ab49900), and donkey anti-goat (Santa Cruz Biotechnologies, sc-2020).

### Luciferase reporter assays

Cells were infected with VEEV-TC83luc at a MOI of 1 in supplemented DMEM for one hour. Luminescence was measured as an indication of viral replication using Promega’s BrightGlo Luciferase Assay System (E2610). The assay was performed according to the manufacturer’s protocol using white-walled, ninety-six well plates (Corning, 3610) seeded with 10,000 cells/well at 16 hpi, unless stated otherwise. Beckman Coulter’s DTX880 Multimode Detector measured luminescence after 100ms integration per well. CC_50_ and EC_50_ were determined by Microsoft Excel or GraphPad Prism v. 6. Nonlinear regression lines were fitted to the data then transformed using the EC_50_ calculator.

### Viral serial passaging in the presence of DMSO or KPT-185

Vero cells were pre-treated with DMSO or KPT-185 (2.5 μM), infected with VEEV TC83 (MOI 0.1), and post-treated with compounds. Supernatants containing infectious virus were collected 1 day post infection, titered (morphology of plaques noted), and used to infect a new set of Vero cells at an MOI of 0.1. VEEV-TC83 was continuously exposed to either DMSO or KPT-185 and passaged 10 times in this manner. Viral supernatants were frozen for every passage and cells were fixed, for capsid localization microscopy analysis, at every other passage beginning with passage 2. At the end of the experiment, a fresh set of Vero cells were infected with viral supernatants (MOI 0.1) from passages 1, 6, and 10. Total RNA was harvested at 6 hpi to isolate those viral genomes that retained infectivity. RNA was isolated utilizing the RNeasy Mini kit (Qiagen) according to manufacturer’s directions. One step reverse transcription (RT)-PCR was performed with 100 ng of total RNA to generate amplicons for sequencing. The SuperScript III One-Step RT-PCR system with Platinum Taq DNA Polymerase (ThermoFisher) was utilized to amplify a 1545 bp fragment encompassing capsid from nucleotides 6970 (nsP4) to 8514 (E3). The following cycling conditions were used: cDNA synthesis, 52°C for 30 min; heat denature, 94°C for 2 min; 40 PCR cycles, 94°C for 15 sec, 55°C for 30 sec, 68°C for 2 min; final extension, 68°C for 5 min. PCR products were then agarose gel purified (MinElute Gel Purification kit, Qiagen) and sequenced.

### Plasmid constructs

The KPT-185 adapted mutations in capsid (T41I, K64E, and K64M) were generated in the pTC83 plasmid. These plasmids and pTC83_Cm, previously described elsewhere [38], were cloned by PCR overlap extension using standard recombinant DNA techniques. All constructs were confirmed by restriction enzyme digestion and sequencing. Plasmid and primer sequences are available upon request.

### Statistical analysis

Unless noted otherwise, mean values were compared using the unpaired, two-tailed student’s t-test available on QuickCalcs, GraphPad’s free online software.

## Results

### Treatment with SINE compounds altered capsid localization

**Fig 1:**
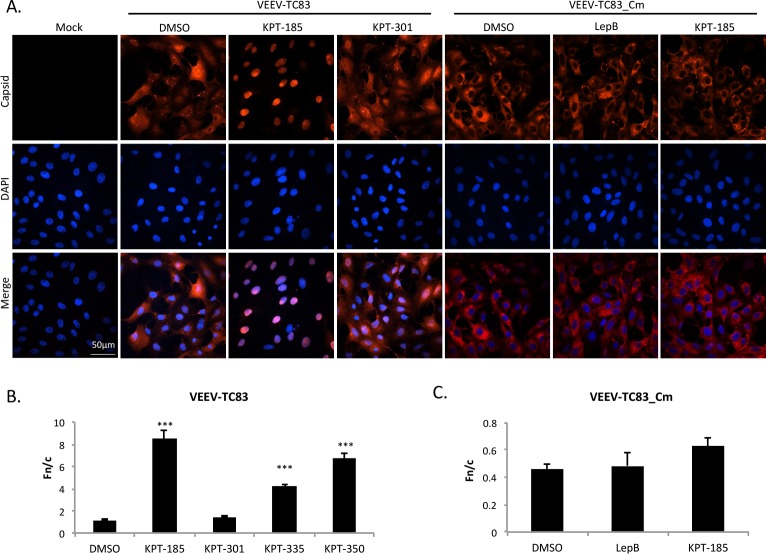
Treatment with SINE compounds altered capsid localization in VEEV-TC83 infected cells. (A) Vero cells were pre-treated with KPT-185 (2.5 μM), KPT-301 (2.5 μM), Leptomycin B (45 nM), or DMSO (0.1%) for two hours prior to infection with VEEV-TC83 or VEEV-TC83_Cm at a multiplicity of infection (MOI) of 1. Cells were post-treated after infection as well. Mock cells were left untreated and uninfected. At 16 hours post-infection (hpi), cells were fixed and probed for capsid (red) and DAPI stained (blue). Data are representative of at least three separate images per treatment group. The scale bar represents 50 μm, with each image captured at the same resolution. (B) Nuclear to cytoplasmic fluorescence ratios (Fn/c) were calculated to quantitate capsid localization. ***p-value ≤ 0.0001 (compared to DMSO treated cells). N is greater than 72 cells. (C) Fn/c values were calculated to quantitate capsid localization, as described in (B). N is greater than 63 cells.

Chemically inhibiting the host’s nuclear import and export proteins alters VEEV capsid localization [18]. Using the same rationale, novel CRM1 inhibitors were tested for their ability to alter capsid localization. Four SINE compounds (S1A Fig) were first screened for toxicity in two separate cell lines, treated for 24 hours, and determined to be non-toxic at 2.5 μM (S2 Fig, [39, 40]). Next, Vero cells treated with non-toxic concentrations of active CRM1 inhibitors (KPT-185, KPT-335, KPT-350), inactive KPT-301, or DMSO were infected with VEEV-TC83, and capsid localization assessed at 16 hpi by confocal microscopy. As seen previously [17, 18], VEEV capsid localized to both the cytoplasm and nucleus in DMSO-treated cells. A similar phenotype was seen upon treatment with the inactive compound, KPT-301. The three active compounds, KPT-185, KPT-335, and KPT-350, confined capsid to the nucleus (Fig 1A and S3A Fig), yielding a localization pattern similar to that seen with treatment by the well-documented CRM1 inhibitor, Leptomycin B [18]. As a control, TC83 virus with a mutated capsid that is defective in nuclear import (TC83_Cm; [38]) was analyzed. TC83_Cm infected cells treated with DMSO, Leptomycin B, or KPT-185 all displayed capsid cytoplasmic staining (Fig 1A). To quantitate capsid localization, the nuclear to cytoplasmic fluorescence ratio (Fn/c) was measured as described previously ([18] and S1B Fig). An Fn/c value greater than one denotes predominately nuclear fluorescence, whereas a score below one indicates predominantly cytoplasmic fluorescence. Treatment with the three active compounds, KPT-185, KPT-335, and KPT-350 had statistically enhanced nuclear accumulation of capsid and Fn/c values of greater than 4 (Fig 1B). In contrast, TC83_Cm infected cells treated with DMSO, Leptomycin B, or KPT-185 had Fn/c values of less than one (Fig 1C).

**Fig 2:**
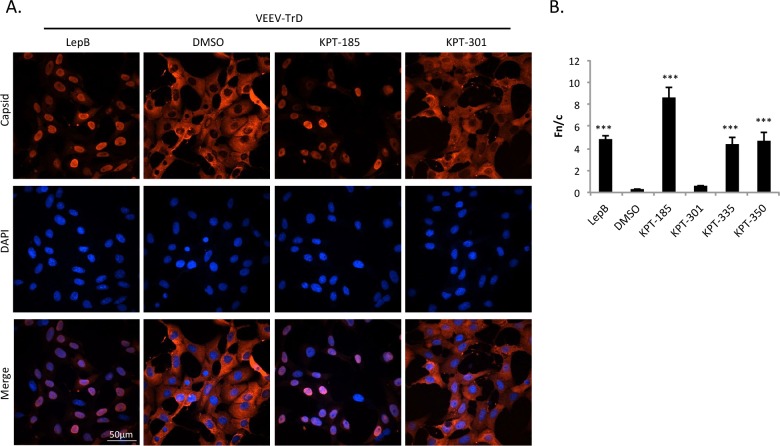
Treatment with SINE compounds altered capsid localization in VEEV-TrD infected cells. (A) Vero cells were pre-treated with 2.5 μM of KPT-185, KPT-301, Leptomycin B (45 nM), or DMSO (0.1%) for two hours prior to infection with VEEV-TrD at a MOI of 1. Cells were post-treated after infection as well. At 16 hpi, cells were fixed and probed for capsid (red) and DAPI-stained (blue). Data are representative of at least three separate images per treatment group. The scale bar represents 50 μm, with each image captured at the same resolution. (B) Fn/c values were calculated to quantitate capsid localization. ***p-value ≤ 0.0001 (compared to DMSO treated cells). N is greater than 49 cells.

To ensure the results seen with VEEV-TC83 mimicked a wild-type infection, SINE compounds were tested against the fully virulent VEEV-TrD strain. Once again, DMSO and inactive KPT-301 treatment had little effect on capsid localization. The active compounds, KPT-185, KPT-335, and KPT-350, like Leptomycin B, resulted in capsid localizing predominately to the nucleus (Fig 2A and S3B Fig) and was statistically distinct from that of DMSO treated controls (Fig 2B). These results indicate that inhibition of CRM1 with the SINE compounds confined both attenuated and wild-type VEEV capsid to the cell nucleus.

### SINE compounds reduced released viral RNA and capsid, indicating interference with viral assembly and/or release

**Fig 3:**
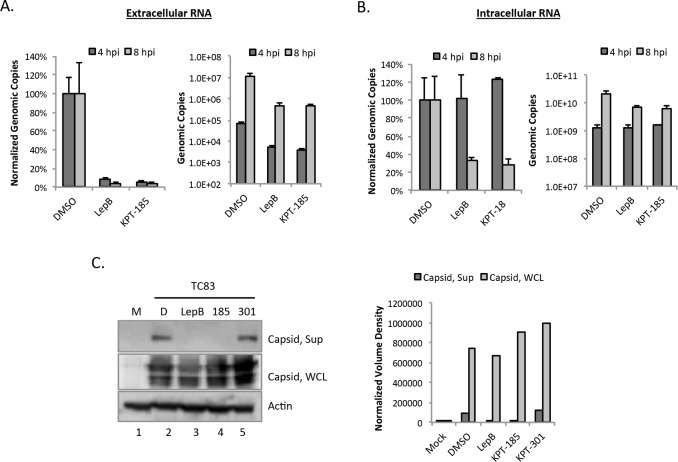
SINE compounds reduced released viral RNA and capsid. (A) Vero cells were pre-treated for two hours with DMSO (1%), Leptomycin B (45 nM), or KPT-185 (2.5 μM) prior to infection with VEEV-TC83 (MOI 1). Cells were post-treated after infection as well. At 4 and 8 hpi, supernatants were collected and extracellular viral RNA extracted and analyzed by q-RT-PCR. Graphs represent biological triplicates and each experiment was performed twice. Genomic copies were normalized as a percentage of the DMSO control (left panel) or shown without normalization (right panel). (B) Vero cells were treated as described above, and total intracellular RNA was extracted from lysed cells and analyzed by q-RT-PCR. Genomic copies were normalized as a percentage of the DMSO control (left panel) or shown without normalization (right panel). (C) Vero cells were treated and infected as described in panel A. At 22 hpi, supernatants (Sup) and whole cell lysates (WCL) were collected. Supernatants were purified using a sucrose gradient. Purified supernatants and WCL were assayed using western blotting and probed for capsid and actin as a loading control. M = Mock; D = DMSO. Right panel: Volume densities of supernatant and WCL capsid from the western blot were normalized to actin.

Knowing that treatment with SINE compounds resulted in increased capsid nuclear localization, it was hypothesized that this would deplete the cytoplasmic pool of capsid, thus limiting viral assembly and decreasing the amount of virus produced. To test this hypothesis, the amount of viral RNA present at 4 and 8 hpi, within cells or in the extracellular supernatants, was assessed. Viral RNA detected in the extracellular supernatants would be virion-associated, while intracellular levels would represent viral RNA to be shuttled into viral protein translation, negative-sense strand RNA replication, or encapsidation into particles. The early time points were chosen to correspond to the first round of viral replication. Extracellular viral RNA was reduced upon treatment with Leptomycin B and KPT-185 at 4 and 8 hpi relative to DMSO controls (Fig 3A), with a greater relative reduction at 8 hpi. In contrast, at 4 hpi, intracellular RNA levels were approximately equal regardless of treatment, with a decrease seen by 8 hpi in both Leptomycin B and KPT-185 treated cells relative to DMSO controls (Fig 3B). Similar results were obtained at an MOI of 10 (S4 Fig). This indicates that SINE compounds likely disrupt a late step in the virus lifecycle such as viral assembly or budding.

To further validate that SINE compounds interfere with virus assembly or budding, the amount of released capsid was compared to intracellular capsid by western blot. Vero cells were treated with SINE compounds or DMSO as described, then infected with VEEV-TC83. Media containing compounds or DMSO were replaced and extracellular supernatants and whole cell lysates were collected 22 hpi. Supernatants were purified by sucrose gradient centrifugation [38]. Capsid was present in the supernatants of DMSO and KPT-301 controls, but not in Leptomycin B or KPT-185 samples. Capsid levels were also unaffected in whole cell lysates from DMSO and KPT-301 treated cells and minimally affected in Leptomycin B or KPT-185 samples (Fig 3C). Thus SINE compounds were not inhibiting viral protein synthesis. These data combined with the altered capsid localization and the observed decrease in viral RNA in extracellular supernatants, indicate that SINE compounds can sequester capsid, removing it from the pool of available structural proteins, leading to decrease in virus assembly and/or release of mature virions.

### Treatment with SINE compounds reduced VEEV replication in a dose-dependent manner

**Fig 4:**
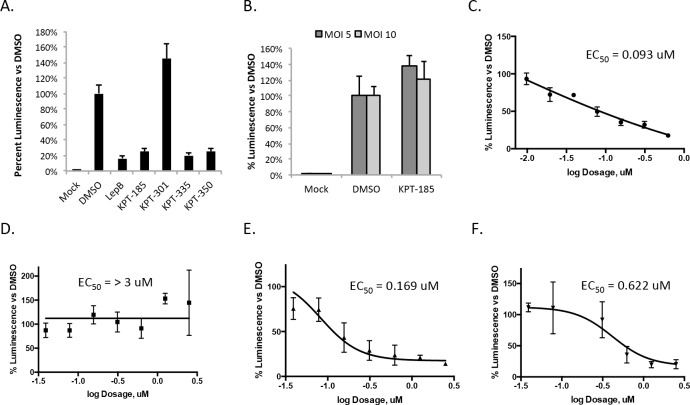
SINE compounds reduced VEEV viral replication in a dose-dependent manner. (A) For two hours prior to infection Vero cells were treated with 2.5 μM of KPT-185, KPT-301, KPT-335, KPT-350, DMSO (1%), or 45 nM Leptomycin B. Cells were infected with VEEV-TC83luc (MOI 1), and cells post-treated after infection. At 16 hpi, the BrightGlo Luciferase Assay was performed. Data are the averaged luminescence of biological triplicates represented as a percentage of luminescence normalized to DMSO-treated cells. The data are representative of two independent experiments. (B) Vero cells were treated as described in panel A and VEEV-TC83luc (MOI 5 or 10) was used for the infection. At 8 hpi, Promega’s BrightGlo Luciferase Assay was performed according to the manufacturer’s protocol. The graph represents percent luminescence compared to DMSO. To calculate EC_50_ values, Vero cells were pre-treated for two hours with 1:2 serial dilutions of KPT-185 (C), KPT-301 (D), KPT-335 (E), or KPT-350 (F), then infected with VEEV-TC83luc (MOI 1) for one hour, cells post-treated with inhibitors, and the BrightGlo Luciferase Assay performed at 16 hpi. Biological triplicates of each concentration were averaged then presented as a percentage of luminescence compared to 1% DMSO-treated cells. The data are representative of two independent experiments.

Since CRM1 inhibitors altered capsid localization and previous work has demonstrated a concurrent reduction in viral replication [18], SINE compounds were examined for their influence on VEEV replication. VEEV-TC83luc, which contains the firefly luciferase gene cloned downstream of a duplicated subgenomic promoter [34], was used as a reporter of viral replication. The active compounds, KPT-185, KPT-335, and KPT-350, and Leptomycin B reduced luminescence at 16hpi by approximately 80%, indicating treatment significantly decreased viral replication (Fig 4A). The inactive compound KPT-301 did not have any effect on viral replication. Importantly, KPT-185 was unable to reduce luminescence when cells were infected at a higher MOI (MOI of 5 or 10) and assayed at 8 hpi (Fig 4B). These data further support the hypothesis that SINE compounds inhibit viral assembly and/or budding.

Using the luciferase assay, the EC_50_, the effective concentration of a compound that reduces luminescence to half that of the DMSO control, was determined. The inactive compound, KPT-301, had an EC_50_ value of >3 μM (Fig 4D), but the three active compounds, KPT-185 (Fig 4C), KPT-335 (Fig 4E) and KPT-350 (Fig 4F) had EC_50_ values that ranged from 0.62 to 0.09 μM (Table 1). The CC_50_ for all four compounds was greater than 10 μM and therefore 10 μM was used to calculate the selective index (SI) for each compound. All three active compounds had SI values greater than 10, indicating the compounds are good therapeutic candidates. This set of experiments demonstrated the active SINE compounds inhibited VEEV replication in a dose-dependent manner with SI values that make them good therapeutic candidates.

**Table 1:** Effective concentrations (50 and 90), cytotoxic concentration (50), and selective indices of SINE compounds against VEEV-TC83luc.

Compound	EC_50_ (μM)	EC_90_ (μM)	CC_50_ (μM)	SI*
KPT-185	0.09	0.89	>10	>111.1
KPT-301	> 3.0	> 3.0	>10	NA
KPT-335	0.17	2.06	>10	>58.8
KPT-350	0.62	2.88	>10	>16.1

*SI = CC_50_ divided by EC_50_

### SINE compounds reduced VEEV-TC83, TrD, but not TC83_Cm titers

**Fig 5:**
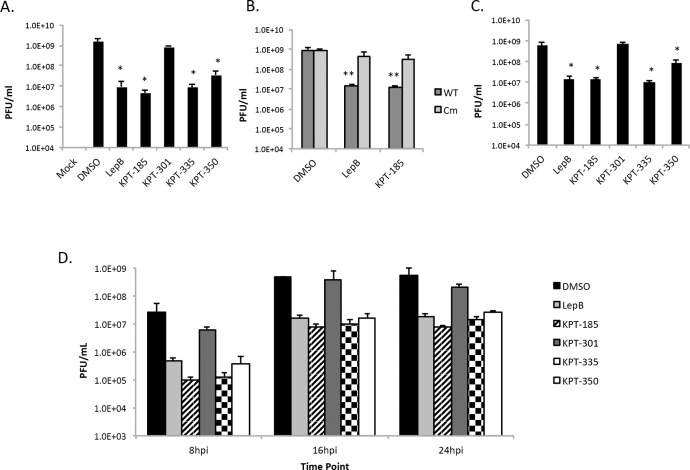
SINE compounds reduced VEEV-TC83, TrD, but not TC83_Cm titers. (A) Vero cells were treated for two hours prior to infection with DMSO (0.5%), 45 nM Leptomycin B, or 2.5 μM of KPT-185, KPT-301, KPT-335, or KPT-350. After an hour infection with VEEV-TC83 (MOI 1), the cells were post-treated with compounds. At 16 hpi, supernatants were collected and plaque assays performed using Vero cells. Graphs represent biological triplicates and each experiment was performed twice. (B) Vero cells were treated as described above and infected with WT TC83 or TC83_Cm (MOI 1). (C) Vero cells were treated as described above and infected with VEEV-TrD (MOI 1). (D) Vero cells were treated as described above and infected with VEEV-TC83 (MOI 1), with supernatants collected at 8, 16, and 24 hpi for plaque assays using Vero cells. Graphs represent biological triplicates. *p-value ≤ 0.05 and ** p-value ≤ 0.01 (compared to DMSO treated cells at the corresponding time point).

As the SINE compounds reduced viral replication of the reporter virus, experiments were performed to confirm the inhibition results using an unmodified virus. Supernatants from TC83 infected cells were collected after 16 hpi and viral titers calculated using plaque assays. The active SINE compounds, KPT-185, KPT-335, and KPT-350, as well as Leptomycin B, were able to significantly reduce TC83 viral titers by approximately two logs (Fig 5A). As a control, cells were alternatively infected with TC83_Cm. Minimal differences in TC83_Cm titers were observed in the presence of Leptomycin B or KPT-185 (Fig 5B). Additionally, active SINE compounds reduced VEEV-TrD viral titers by approximately two logs (Fig 5C). Conversely, treatment with inactive KPT-301 yielded viral titers similar to that observed for DMSO alone (Fig 5A and 5C). Performing a similar experiment with supernatant collected at 8, 16, and 24 hpi with TC83 yielded results analogous to the 16 hpi experiments (Fig 5D), indicating the SINE compounds have an effect early on infection as well as at later time points.

### SINE compounds altered capsid localization and reduced viral titers when used as a post-treatment only

**Fig 6:**
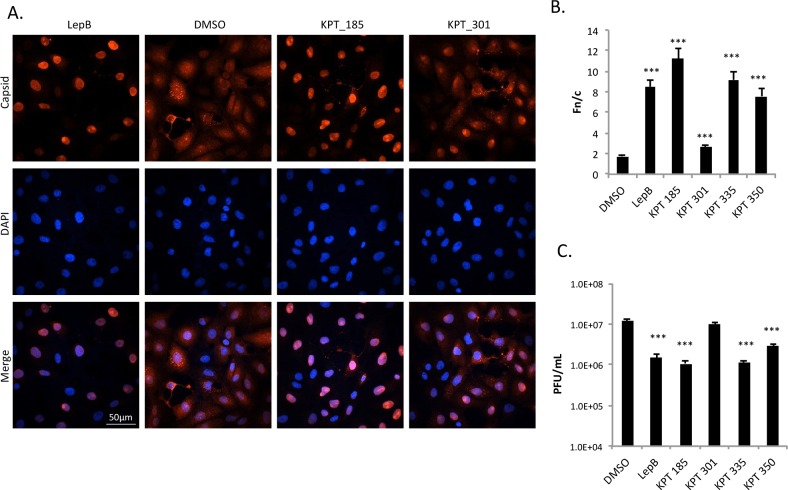
SINE compounds altered capsid localization and reduced viral titer when used as a post-treatment only. (A) Vero cells were infected with VEEV-TC83 (MOI 1) for one hour. Media without drugs was added after infection. Four hours later, cells were post-treated with 2.5 μM of KPT-185, KPT-301, or DMSO (0.1%) for four hours. At 8 hpi, cells were fixed and probed for capsid (red) and DAPI stained (blue). The scale bar represents 50 μm, with each image captured at the same resolution. (B) Fn/c values were calculated to quantitate capsid localization. Treatment with the three active compounds, KPT-185, KPT-335, and KPT-350 had statistically significant accumulations of nuclear fluorescence, as comparable with Leptomycin B treatment. *** p-value ≤ 0.0001 (compared to DMSO treated cells). N is greater than 50 cells. (C) Vero cells treated as in (A), collected at 8 hpi, and plaque assays performed using Vero cells. Graphs represent biological triplicates and each experiment was performed twice. *** p-value ≤0.0001 (compared to DMSO treated cells).

All of the previous experiments involved pre- and post-treating cells with SINE compounds; however, treatment of cells after infection is more predictive of a compound’s therapeutic potential. To this end, Vero cells were infected with VEEV-TC83 for one hour, viral inocula removed, and cells washed prior to addition of media. After four hours, active SINE compounds, the inactive compound KPT-301, Leptomycin B, or DMSO was added to the media. At 8 hpi capsid localization and VEEV titers were determined. The active SINE compounds and Leptomycin B (Fig 6A and 6B and S3C Fig) all showed capsid localization patterns similar to that seen with pre- and post-treated cells (Fig 1). Post-treatment with DMSO had no effect on capsid localization, while KPT-301 (Fig 6A and 6B) had a slight but statistically significant effect on capsid localization. However, this effect was much less dramatic as compared to the active SINE compounds. Similarly, post-treatment of Vero cells infected with VEEV-TC83 significantly reduced viral titers by approximately one log (Fig 6C). Together, these results indicate that active SINE compounds may have therapeutic, in addition to prophylactic, potential.

### Interferon responsive genes are induced in the presence of SINE compounds

**Fig 7:**
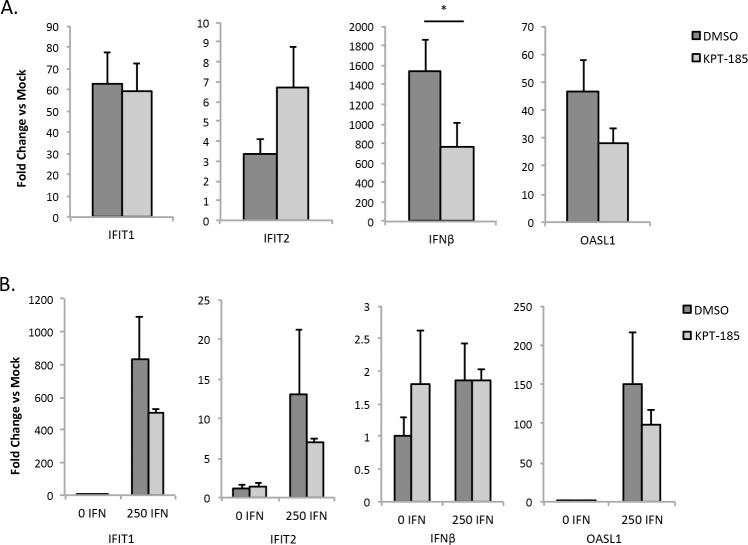
Interferon stimulated genes are induced in the presence of SINE compounds. (A) MEFs were pre-treated with DMSO or KPT-185 (2.5 μM) for two hours, infected with VEEV-TC83 (MOI 1), and post-treated following infection. Mock infected cells were processed alongside as controls. RNA lysates were prepared at 16 hpi. qRT-PCR was performed using TaqMan Gene Expression Assays for IFIT1, IFIT2, IFNβ, and OASL1. 18S rRNA was used as the endogenous control. *p-value < 0.05. (B) MEFs were pre-treated as described in (A). After pre-treatment, 0 or 250 IU interferon-β was added, and RNA lysates were prepared four hours later. qRT-PCR was performed as described in (A).

Type I interferon signaling is important for the innate immune response to VEEV infection. As such, SINE compounds were examined for their influence on interferon stimulated genes (ISGs) following VEEV-TC83 infection. Interferon competent MEFs were selected for this analysis. Four ISGs, IFIT1, IFIT2, IFNβ, and OASL1, were analyzed following infection in the presence of DMSO or KPT-185. These four genes were selected as they have previously been shown to be induced following VEEV infection [41, 42]. All four transcripts were strongly induced following TC83 infection (Fig 7A). Cells treated with KPT-185 also displayed increased levels of all four transcripts, with only IFNβ being significantly reduced compared to DMSO treated cells. SINE compounds were also examined for their influence on these genes in the absence of infection (Fig 7B). While there was a slight reduction of IFIT1, IFIT2, and OASL1 expression in KPT-185 treated cells as compared to DMSO after interferon addition, this was not statistical significant. These results indicate that ISGs are capable of being induced in the presence of SINE compounds.

### SINE compounds reduced viral titers of other New World alphaviruses

**Fig 8:**
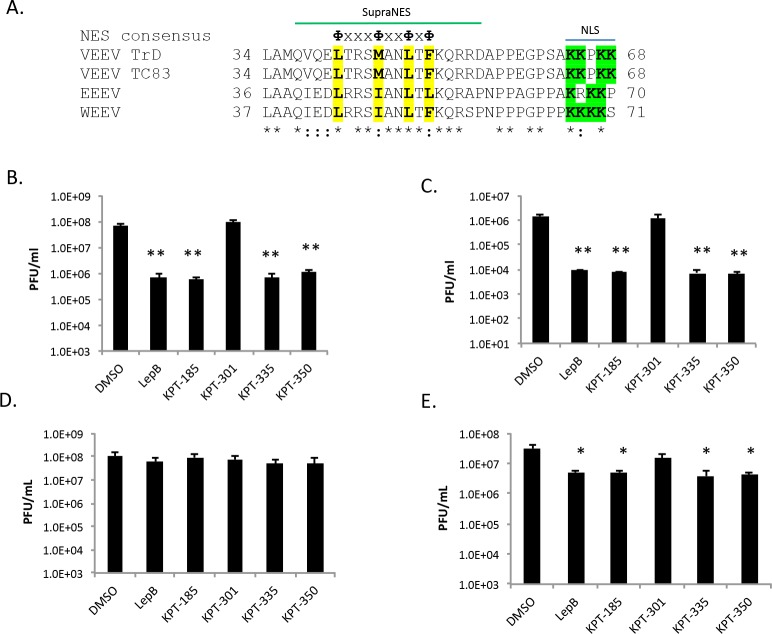
SINE compounds inhibited New World alphaviruses more dramatically than Old World alphaviruses. (A) Capsid alignment using Uniprot. Asterisks indicate positions which have a fully conserved residue. Colons indicate conservation between amino acids of strongly similar properties. Periods indicate conservation between amino acids of weakly similar properties. Residues within the consensus NES sequence (ФxxxФxxФxФ, where Ф = L, I, F, V or M and x = any amino acid) are highlighted in yellow. Positively charged residues within the NLS are highlighted in green. The supraNES sequence identified in VEEV capsid is indicated [17]. Conservation among different strains of the four viruses aligned—VEEV-TrD (GenBank Accession Number AAB0251), VEEV-TC83 (CAA27883), EEEV-82V-2137 (CAA29261), and WEEV-BFS1703 (AAA42999)–was strong for the sequence examined. (B) Vero cells were treated for two hours prior to infection with DMSO (0.5%), 45 nM Leptomycin B, or 2.5 μM of KPT-185, KPT-301, KPT-335, or KPT-350. After an hour infection with EEEV (GA97) (MOI 1), the cells were post-treated with inhibitors. At 16 hpi, supernatants were collected and plaque assays performed using Vero cells. Graphs represent biological triplicates and each experiment was performed twice. (C) Vero cells were treated as described above, infected with WEEV (California 1930) (MOI 1), and titered by plaque assay in Vero cells. (D) Vero cells were treated as described above, infected with SINV (EgAr 339) (MOI 1), and plaque assays were performed using BHK-21 cells. (E) Vero cells were treated as described above, infected with CHIKV (S27) (MOI 1), and plaque assays were performed using BHK-21 cells. *p-value ≤ 0.001, **p-value ≤ 0.005 (compared to DMSO treated cells at the corresponding time point).

It was hypothesized that the SINE compounds should have a similar effect on other New World alphaviruses, assuming their capsid proteins also gain access to the nucleus and interact with CRM1 to egress from the nucleus [19, 21]. Frolova’s group demonstrated that VEEV capsid contains an NLS at amino acids 64–68 and a supraNES in the region of 38–55 [17]. Using UniProt, the capsid proteins of several New World alphaviruses were aligned. The capsid proteins of VEEV, EEEV, and WEEV share critical lysine residues [17] in the positively charged NLS (Fig 8A, residues highlighted in green). A consensus NES sequence (ФxxxФxxФxФ, where Ф = L, I, F, V or M and x = any amino acid) is present in VEEV, EEEV, and WEEV capsid proteins ([17], Fig 8A). A common NES region would suggest that capsid proteins from other New World alphaviruses would interact with CRM1 in a similar manner. To test this hypothesis, SINE compounds were examined for their ability to inhibit EEEV and WEEV. A statistically significant inhibition of approximately two logs was seen with the active SINE compounds for both EEEV (Fig 8B) and WEEV (Fig 8C), while inactive KPT-301 yielded similar results to DMSO alone. This suggests that the active SINE compounds are likely acting on a common mechanism among the New World alphaviruses. Similar experiments were performed with the Old World alphaviruses, SINV and CHIKV. No inhibition was seen with SINV (Fig 8D), but there was a small and statistically significant difference seen with CHIKV (Fig 8E). It can thus be concluded that inhibition of CRM1 has a more pronounced effect on New World alphaviruses.

### Multiple-passages in the presence of KPT-185 induced mutations in VEEV-TC83 capsid

**Fig 9:**
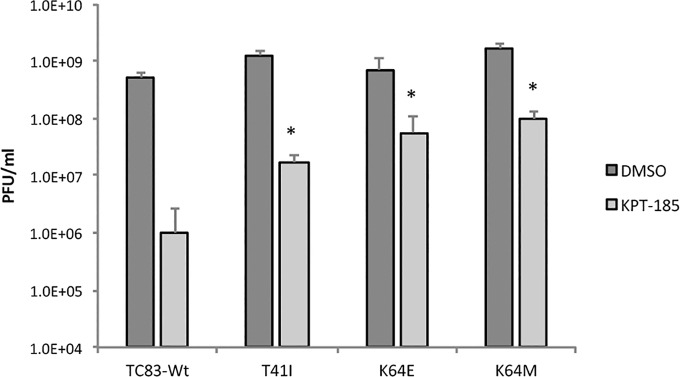
Capsid mutations confer some resistance to SINE treatment. Vero cells were pretreated with DMSO (1%) or KPT-185 (2.5 μM) for two hours then infected with TC83-Wt, TC83-T41I, TC83-K64E, or TC83-K64M at a MOI of 1 for one hour. Cells were washed with 1X PBS and media containing either DMSO or KPT-185 added. Supernatants were collected 16 hpi, and titers determined by plaque assay using BHK-21 cells. Graphs represent biological triplicates. *p-value ≤ 0.05 (compared to KPT-185 treated cells infected with TC83-Wt).

To determine if adaptive mutations within the VEEV capsid open reading frame (ORF) could occur in the presence of the CRM1 inhibitor, VEEV-TC83 was serially passaged ten times in the presence of DMSO or KPT-185. Viral titers and plaque morphology for three replicates at each passage are presented in S1 Table. Capsid localization was assessed every other passage by confocal microscopy (S5 and S6 Figs). Plaque morphology, viral titers, and capsid localization of DMSO treated samples stayed consistent during passages 1–9. At passage (P)10, two of the three DMSO treated replicates displayed smaller punctate plaque morphology. For KPT-185 treated samples, starting at P5 and 6 for KPT-1 and KPT-2, respectively, plaques became cloudy and reminiscent of plaques formed by TC83_Cm virus. No changes in plaque morphology were observed with KPT-3. By P10 an increase in viral titers was observed for all three KPT replicates, suggesting that KPT-185 resistant viruses had developed. With respect to capsid localization, at P2, all KPT replicates had a mixed phenotype with either predominantly nuclear or cytoplasmic staining. However, by P6, all KPT replicates displayed predominantly cytoplasmic localization that was maintained for the remainder of the passages. Sequencing of the capsid ORF from viruses obtained from passages 1, 6, and 10 was performed for all replicates to determine what adaptive mutations had occurred (Table 2). No changes were found in the capsid ORF in TC83 passaged in the presence of DMSO. In contrast, all three replicates of TC83 passaged in the presence of KPT-185 displayed substitutions within the NLS or NES motifs. By P10, KPT-1 and KPT-2 both acquired a K64 mutation located within the NLS, disrupting positive charge at this position. The K64M mutation for the KPT-1 replicate was detected at P6 when plaque morphology was cloudy and capsid localization became largely cytoplasmic. In contrast, the KPT-2 replicate acquired a three amino acid deletion (^61^PSA) that was not stably maintained within the population and a K64E mutation was detected instead at/by P10. Finally, for the KPT-3 replicate, which maintained normal plaque morphology, but did have altered capsid staining, a mutation within capsid’s NES motif, T41I, was detected at P10. Next, to assess whether these mutations conferred KPT-185 resistance, three capsid mutated viruses (T41I, K64E, and K64M) were constructed. Cells pre- and post-treated with KPT-185 or the vehicle were infected with either wild-type TC83 (TC83-Wt) or one of the three mutated capsid viruses (Fig 9). All three capsid mutants were significantly resistant to KPT-185 treatment as compared to TC83-Wt, indicating that these mutations were at least partially responsible for the observed resistance after KPT-185 passaging. Collectively, these results demonstrate that maintenance of VEEV in the presence of the CRM1 inhibitor, KPT-185, can result in adaptive mutations that alter capsid localization and result in drug resistance.

**Table 2:** Sequence analysis of VEEV capsid ORF.

Passaged Replicate	KPT-1^*d*^			KPT-2			KPT-3		
Passage Number	1	6	10	1	6	10	1	6	10
Nucleotide change^*a*^		A7752T	A7752T		del 7742–7750	A7751G			C7686T
Amino acid change^*b*^		K64M	K64M		del 61–63^*c*^	K64E			T41I

^*a*^Nucleotide numbering is based on TC83 reference (Genbank Accession # L01443)

^*b*^Amino acid numbering is capsid-specific

^c^Three amino acid deletion is P-S-A

^*d*^TC83 passaged in the presence of DMSO did not yield any changes within the capsid ORF

## Discussion

CRM1, which regulates nuclear export of >200 proteins, including many viral proteins, has surfaced recently as an attractive anti-viral host target [29]. CRM1 is essential for some viruses’ life cycles through regulation of viral protein nuclear export. Karyopharm Therapeutics has developed a new class of CRM1 inhibitors, SINE compounds, with drugs from this class proving to be well tolerated and active in the clinic for canine and human cancers. In addition to the potent anticancer activity of these compounds, they have been shown to have substantial anti-inflammatory and neuroprotective activity *in vitro* and *in vivo* resulting from CRM1 inhibition. CRM1 mediates nuclear export of a variety of proteins important for inflammation, including the NF-κB pathway regulatory proteins IκBα, IκBε, RelA, p100, as well as COMMD1, HSCARG, Forkhead Box, and Nrf2 transcription factors, RXRα and PPARγ nuclear receptors and the chromatin binding protein HMGB1 [43–51]. It has been demonstrated that forced nuclear retention of many of these CRM1 protein cargos with the SINE compound KPT-350 leads to anti-inflammatory and neuroprotective effects [52]. SINE compounds also decreased expression of the pro-inflammatory cytokines IFN-γ, IL-1β, IL-6 and TNF-α in influenza A H1N1-infected mouse and ferret lungs [30]. Finally, toxicological studies in rats and monkeys have been initiated in preparation for clinical development. Many alphaviruses including VEEV manipulate the same pathways, as reviewed by Steele [53] and Suhrbier [54]. We have previously shown that chemically inhibiting modulators of pro-inflammatory cytokines reduces VEEV replication cycles and has some neuroprotective outcomes [35]. Future studies will examine the anti-apoptotic, neuroprotective, and anti-inflammatory qualities of the SINE compounds in the context of an alphavirus infection.

VEEV is often used as a model organism for New World alphavirus research. We have previously demonstrated the necessity of the CRM1/VEEV capsid interaction through siRNA silencing of CRM1 [18]. Here we have demonstrated that SINE compounds alter VEEV capsid localization, disrupting viral assembly and reducing overall viral levels. Based on EEEV and WEEV sharing conserved residues within the capsid NES, we hypothesized that the mechanism of interaction between host and viral proteins is common to New World alphaviruses. Under that assumption, it was demonstrated that EEEV and WEEV could also be inhibited using SINE compounds. This suggests that the CRM1/capsid interaction is important for the replication of New World alphaviruses, and disrupting it through chemical inhibition most likely touches upon a common virus/host interaction.

In contrast, nsP2 of Old World alphaviruses, such as SINV, have been shown to localize to the nucleus and induce transcriptional shutoff as opposed to capsid [10, 55]. This is achieved in part through nsP2-mediated ubiquitination of Rpb1, a subunit of the RNA polymerase II complex, leading to its degradation in both SINV and CHIKV infections [56]. A recent study by the Herchenröder group identified an NES in capsid at amino acids 143 to 155 for CHIKV [57]. Mutating this sequence confined GFP-tagged capsid to the nucleus, as did treatment with Leptomycin B [57]. Results from this study are in tentative agreement with the results presented within, as treatment with Leptomycin B and SINE compounds significantly reduced CHIKV, but not SINV titers. However, Leptomycin B and SINE compounds inhibited New World alphaviruses (VEEV, EEEV, and WEEV) to a greater extent than CHIKV. These results suggest that Old World alphaviruses are either less or not dependent on CRM1 for viral replication. As SINE compounds will also influence host protein nuclear trafficking, the inhibition of viral replication observed could also be due to inhibition of host protein trafficking, instead of viral protein trafficking, or a combination of both. Unfortunately, the lack of commercial antibodies for CHIKV and SINV capsid and nsP2 proteins makes further analysis difficult. Tagging the proteins is beyond the scope of this paper, but invites further consideration for future studies.

Our data suggest the SINE compounds reduce viral titer by interfering with viral assembly and/or budding. The Frolov lab has demonstrated that VEEV virions can assemble directly at the plasma membrane without preassembly of nucleocapsids in the cytoplasm [58]. The amino terminal domain of capsid is dispensable for packaging but is required for RNA encapsidation [58]. Packaging signals on the encapsidated, genomic viral RNA, 4–6 stem loop structures of GGG sequences at the base, are recognized by capsid [59]. Envelope glycoproteins journey from the endoplasmic reticulum to the Golgi network before arriving at the plasma membrane. Capsid associates with the E2 glycoprotein in the plasma membrane in a process extensively studied but still not well understood [60]. From our study, Leptomycin B or KPT-185 treatment resulted in a reduction of extracellular RNA and capsid levels at early time points post infection, while intracellular levels were minimally affected at 8 hpi. This indicates viral RNA replication and translation were unaffected. These data suggest that SINE compound treatment results in accumulation of capsid in the nucleus as opposed to the cytoplasm thus delaying viral genome encapsidation, as well as interfering with capsid/glycoprotein interaction and subsequent virus particle budding.

The emergence of drug resistant mutants as well as the inherent genetic variability of viruses are significant barriers to the prevention and treatment of viral infections that rely solely on directly acting antivirals. The use of inhibitors targeting static cellular factors that are required for viral replication and may affect multiple strains/genotypes has become an attractive possibility for standalone therapies or in combination with antivirals [61–65]. However, this approach has not been without caveats, most notably, that viruses can acquire resistant mutants that circumvent their dependency on these host factors for viral replication [66–68]. How high this barrier to resistance for host-targeting antivirals is, will likely depend on the presence of naturally occurring polymorphisms [68]. From our own studies with passaging VEEV-TC83 in the presence of KPT-185, we observed the development of NES/NLS mutations (K64E, K64M, and T41I) that disrupted the trafficking of capsid to the nucleus and resulted in increased titers. For a nuclear export inhibitor to select for compensatory mutations within the capsid NLS motif as well as the NES T41I mutation to alter capsid nuclear localization suggests that there is a close genetic linkage between the capsid NES and NLS motifs. This linkage is further supported by a prior report where deletion of subdomain 2 (aa 38 to 51) of capsid (VEEV/CmΔ2) containing the NES motif, lead to a compensatory mutation within the NLS domain, K64E [58]. The K64E mutation (VEEV Δ2ad) restored cytoplasmic accumulation of nucleocapsid to levels similar to wildtype VEEV and had increased titers as compared to VEEV/CmΔ2. VEEV capsid forms a tetrameric complex with the host’s nuclear import and export proteins, importin α/β1 and CRM1, which obstructs the nuclear pore complex [17]. Given the close proximity of the NLS and NES motifs in capsid, mutation of either site may alter local secondary structure leading to disruption of this tetrameric complex overall.

Because the capsid protein shares homology [19] between the three New World alphaviruses tested, we hypothesize that SINE compounds may also disrupt viral assembly in WEEV and EEEV infections. Future studies will focus on determining if SINE treatment affects these viruses in a similar manner to VEEV, including examining capsid localization and the effect on viral assembly. It is known that the capsid proteins of VEEV [10, 69], EEEV [10, 20], and WEEV [70] contribute to the inhibition of host transcription, dampening the innate immune response. In addition, EEEV capsid is found in the nucleus at early time points after infection [71]. Therefore, we speculate that modulating nuclear trafficking may be a common mechanism of New World alphavirus capsid proteins to dampen the cellular response to infection and contribute to pathogenesis. Further, if VEEV, WEEV, and EEEV capsid proteins all target the same host proteins, i.e. nuclear import and export proteins, then a common host-targeted therapeutic should be achievable.

## Supporting Information

S1 Fig SINE Compound Table and Example of an Fn/C Calculation. (A) Table containing molecular structures and weights of SINE compounds. (B) The ratio of nuclear (Fn) to cytoplasmic (Fc) fluorescence (Fn/c) is calculated as follows: Fn/c = (Fn-Fb)/(Fc-Fb), where Fb is background autofluorescence. Example images come from previously unpublished experiments and based on a previously published figure [18].
(TIF)

S2 Fig Cytotoxicity curves of SINE compounds. Vero cells were treated with 1:2 serial dilutions of KPT-185 (A), KPT-301 (B), KPT-335 (C), and KPT-350 (D). Luminescence was measured using Promega’s CellGlo Viability Assay using the manufacturer’s protocol at 24 hours post-treatment.
(TIF)

S3 Fig Treatment with KPT-335 and KPT-350 altered capsid localization. (A) Vero cells were pre-treated with 2.5 μM of either KPT-335 or KPT-350 for two hours prior to infection with VEEV-TC83 at a multiplicity of infection (MOI) of 1. Cells were post-treated after infection as well. At 16 hpi, cells were fixed and probed for capsid (red) and DAPI stained (blue). Data are representative of at least three separate images per treatment group. The scale bar represents 50 μm, with each image captured at the same resolution. (B) Same as in panel A except cells were infected with VEEV-TrD. (C) Same as in panel A except cells were post-treated only and collected at 8 hpi.
(TIF)

S4 Fig SINE compounds reduced released viral RNA at MOI of 10. (A and B) Vero cells were pre-treated for two hours with DMSO (1%), Leptomycin B (45 nM), or KPT-185 (2.5 μM) prior to infection with VEEV-TC83 (MOI 10). Cells were post-treated after infection as well. At 4 and 8 hpi, supernatants were collected and extracellular viral RNA extracted and analyzed by q-RT-PCR. Panel A displays the data normalized as a percentage of the DMSO control and panel B as genomic copies. (C and D) Vero cells were treated as described above, and total intracellular RNA was extracted from lysed cells and analyzed by q-RT-PCR. Panel C displays the data normalized as a percentage of the DMSO control and panel D as genomic copies.
(TIF)

S5 Fig Capsid localization in VEEV serially passaged in the presence of DMSO. Vero cells infected with VEEV-TC83 (MOI 0.1) and treated with DMSO were collected at passage 2, 4, 6, and 10. Cells were fixed, probed for capsid (red) and DAPI stained (blue), and imaged using confocal microscopy.
(TIF)

S6 Fig Capsid localization in VEEV serially passaged in the presence of KPT-185. Vero cells infected with VEEV-TC83 (MOI 0.1) and treated with KPT-185 (2.5 μM) were collected at passage 2, 4, 6, and 10. Cells were fixed, probed for capsid (red) and DAPI stained (blue), and imaged using confocal microscopy.
(TIF)

S1 Table Viral titers and plaque morphology of VEEV serially passaged in the presence or absence of SINE. VEEV-TC83 was serially passaged ten times in the presence of DMSO or KPT-185 in triplicate. Viral titers were determined by plaque assay after each passage, and plaque morphology was noted for each replicate. ‘Normal’ indicates plaques that are large, have clear boundaries, and are easily counted. ‘Punctate’ indicates plaques that are small or pinpoint but still countable. ‘Cloudy’ indicates plaques that are cloudy, have diffuse boundaries, and are difficult to count.
(XLSX)
