# Supplementary figures and images for: Selective Inhibitor of Nuclear Export (SINE) Compounds Alter New World Alphavirus Capsid Localization and Reduce Viral Replication in Mammalian Cells
Source: PLoS Negl Trop Dis. 2016 Nov 30;10(11):e0005122. doi: 10.1371/journal.pntd.0005122 (PMC5130180; doi:10.1371/journal.pntd.0005122)

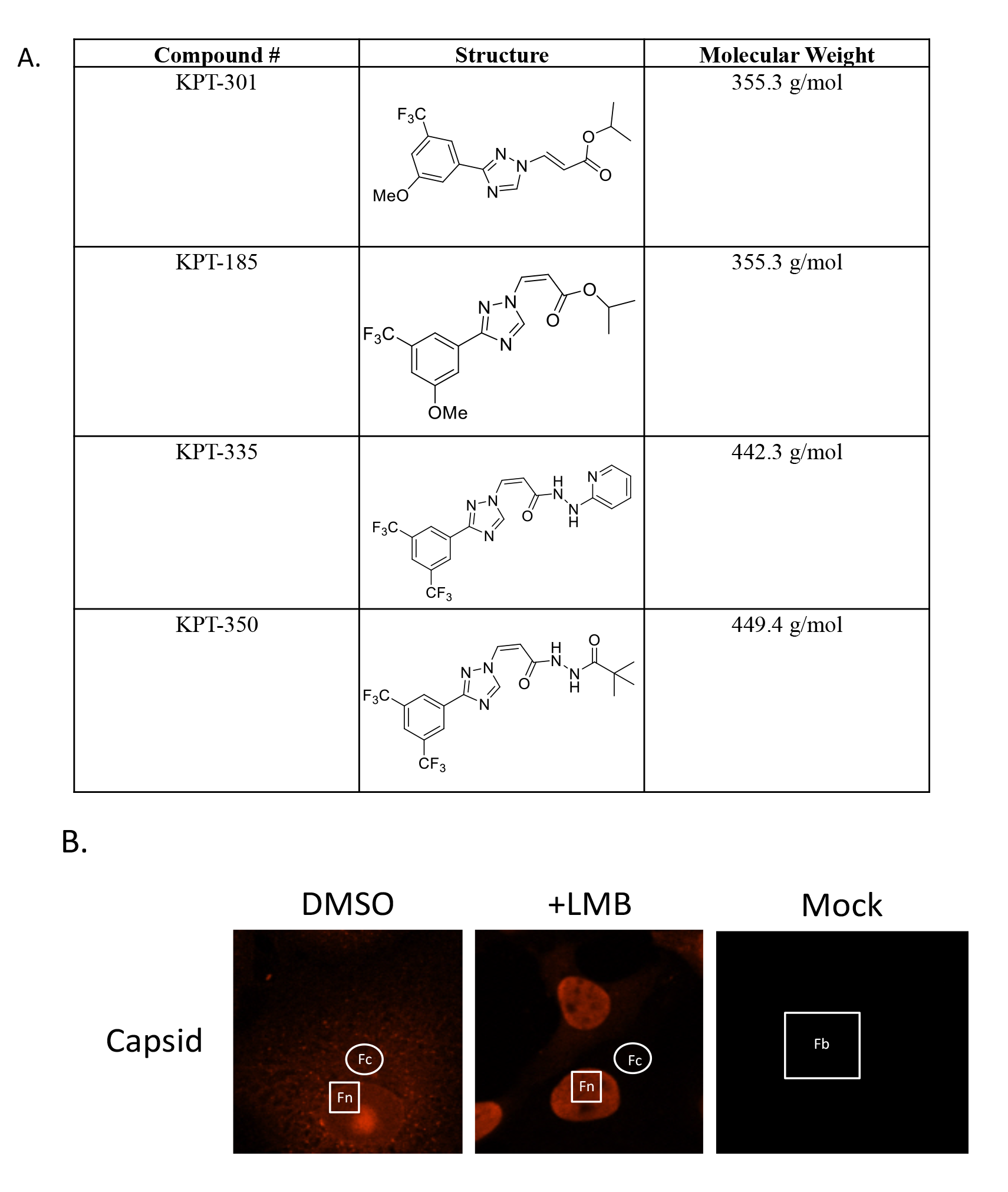

Supplement: S1 Fig — (A) Table containing molecular structures and weights of SINE compounds. (B) The ratio of nuclear (Fn) to cytoplasmic (Fc) fluorescence (Fn/c) is calculated as follows: Fn/c = (Fn-Fb)/(Fc-Fb), where Fb is background autofluorescence. Example images come from previously unpublished experiments and based on a previously published figure [18]. (TIF) [file pntd.0005122.s001.tif]

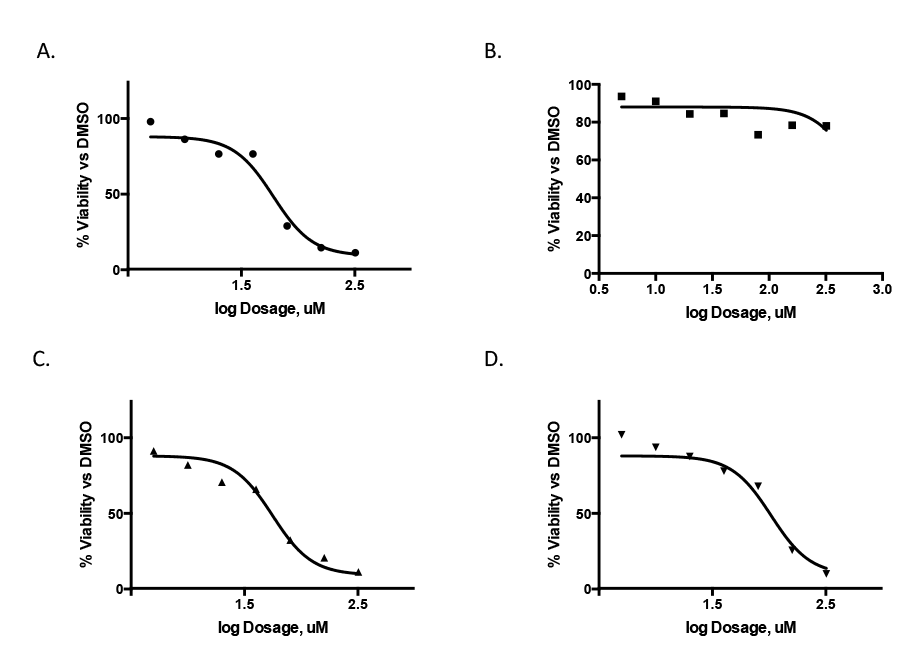

Supplement: S2 Fig — Vero cells were treated with 1:2 serial dilutions of KPT-185 (A), KPT-301 (B), KPT-335 (C), and KPT-350 (D). Luminescence was measured using Promega’s CellGlo Viability Assay using the manufacturer’s protocol at 24 hours post-treatment. (TIF) [file pntd.0005122.s002.tif]

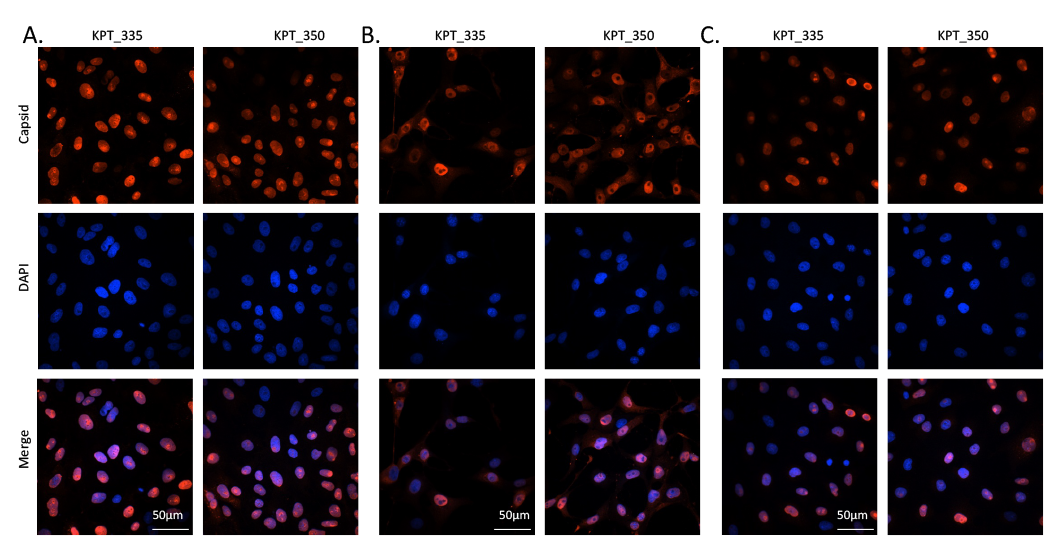

Supplement: S3 Fig — (A) Vero cells were pre-treated with 2.5 μM of either KPT-335 or KPT-350 for two hours prior to infection with VEEV-TC83 at a multiplicity of infection (MOI) of 1. Cells were post-treated after infection as well. At 16 hpi, cells were fixed and probed for capsid (red) and DAPI stained (blue). Data are representative of at least three separate images per treatment group. The scale bar represents 50 μm, with each image captured at the same resolution. (B) Same as in panel A except cells were infected with VEEV-TrD. (C) Same as in panel A except cells were post-treated only and collected at 8 hpi. (TIF) [file pntd.0005122.s003.tif]

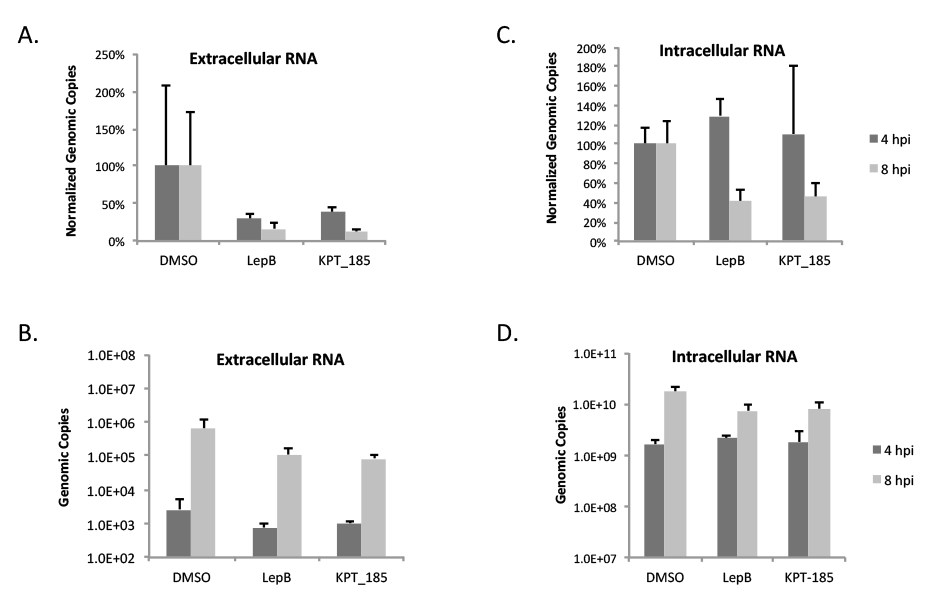

Supplement: S4 Fig — (A and B) Vero cells were pre-treated for two hours with DMSO (1%), Leptomycin B (45 nM), or KPT-185 (2.5 μM) prior to infection with VEEV-TC83 (MOI 10). Cells were post-treated after infection as well. At 4 and 8 hpi, supernatants were collected and extracellular viral RNA extracted and analyzed by q-RT-PCR. Panel A displays the data normalized as a percentage of the DMSO control and panel B as genomic copies. (C and D) Vero cells were treated as described above, and total intracellular RNA was extracted from lysed cells and analyzed by q-RT-PCR. Panel C displays the data normalized as a percentage of the DMSO control and panel D as genomic copies. (TIF) [file pntd.0005122.s004.tif]

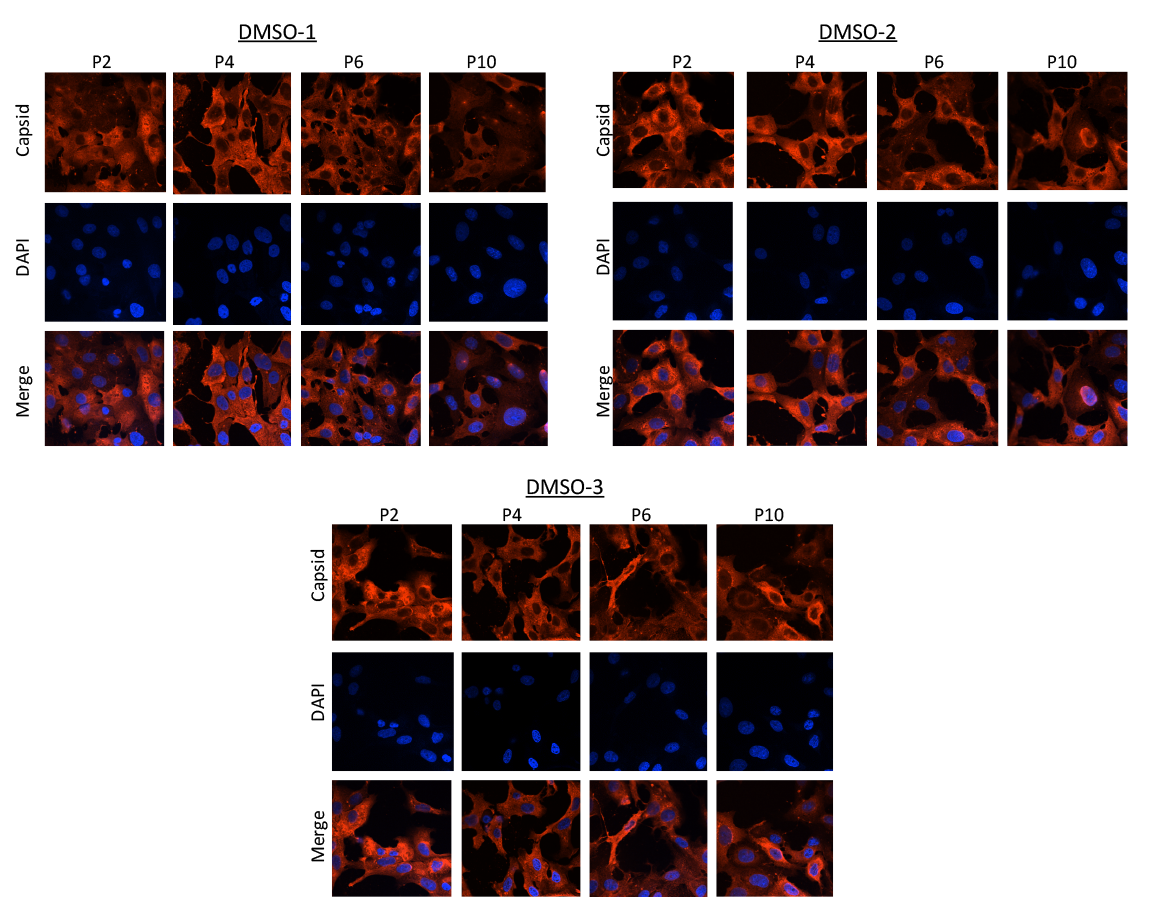

Supplement: S5 Fig — Vero cells infected with VEEV-TC83 (MOI 0.1) and treated with DMSO were collected at passage 2, 4, 6, and 10. Cells were fixed, probed for capsid (red) and DAPI stained (blue), and imaged using confocal microscopy. (TIF) [file pntd.0005122.s005.tif]

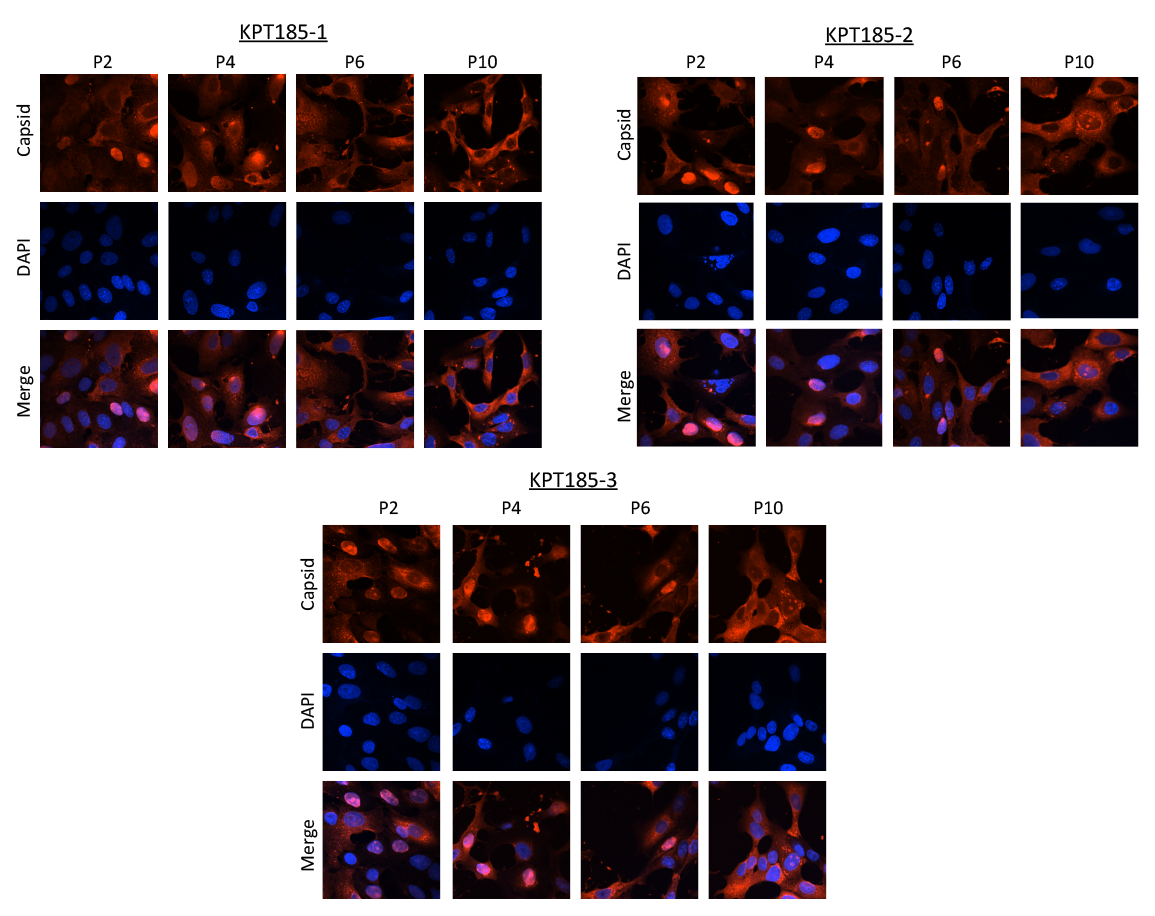

Supplement: S6 Fig — Vero cells infected with VEEV-TC83 (MOI 0.1) and treated with KPT-185 (2.5 μM) were collected at passage 2, 4, 6, and 10. Cells were fixed, probed for capsid (red) and DAPI stained (blue), and imaged using confocal microscopy. (TIF) [file pntd.0005122.s006.tif]
